# New research on media coverage of mental illness and suicide: implications for stigma, suicide mortality and social inclusion.

**DOI:** 10.1192/j.eurpsy.2023.98

**Published:** 2023-07-19

**Authors:** S. Calvo Satorres

**Affiliations:** University of Valencia, Valencia, Spain

## Abstract

**Abstract: Introduction:**

The Werther effect is a phenomenon that has been demonstrated in the media. These media are being replaced by social media, the use of which has increased greatly over the decade especially among young people, allowing content to be streamed instantly and fostering connections around the world. At the same time, there is a deterioration in mental health among young people.

**Objectives:**

To know if there is a Werther effect on social networks, as well as to understand the relationship between this effect and social media. It was also wanted to know if the posts follow the guidelines for dealing with suicide if there are differences between types of social media and if this effect is observed after the death of celebrities.

**Methods:**

A systematic review was performed with analysis, data extraction and synthesis of the structured results following the PRISMA criteria.

**Results:**

15 articles were included, 11 of which showed Werther effects on social media, while only 4 were unrelated. It was found that most publications did not follow the recommended guidelines. No differences were observed between social media, while a greater effect was observed when it was related to celebrity suicide.

**Conclusions:**

Social media can act as a suicide support network, as it is a space where there is a risk of infection by normalizing suicide and treating it insecurely. However, it has also been shown that it can act as an agent of change and protection, as many public individuals on social media seek help and express their concerns. More studies are needed to know the magnitude of the Werther effect on social media and to know the potential benefits of talking about suicide if it is done safely.

**Disclosure of Interest:**

None Declared

